# Acute C4 Ingestion and Toxicity: Presentation and Management

**DOI:** 10.7759/cureus.7294

**Published:** 2020-03-16

**Authors:** Stephen Chong, Brit Long, Joseph K Maddry, Vikhyat S Bebarta, Patrick Ng

**Affiliations:** 1 Emergency Medicine, Brooke Army Medical Center, Fort Sam Houston, USA; 2 Military and Emergency Medicine, Uniformed Services University, Bethesda, USA; 3 Emergency Medicine, University of Colorado Anschutz Medical Campus, Aurora, USA

**Keywords:** rdx, c4, composition 4, explosive, ingestion, toxicity, seizure

## Abstract

Composition C4 is a plastic explosive substance used in military combat units for demolition. The active component of composition C4 is hexahydro-1,3,5-trinitro-1,3,5-triazine, also known as RDX (Royal Demolition Explosive). There are limited reports of the effects of ingested C4. We sought to provide a broad overview of cyclonite/RDX exposure in humans to give a general understanding of both the clinical effects of exposure as well as management considerations. The authors searched MEDLINE and Google Scholar for articles using the keywords for this literature review, including case reports, case series, animal studies, clinical guidelines, and reviews. There are few reports and studies of C4 ingestion. In these studies, the most common effects are serious central nervous system (CNS) effects followed by renal and gastrointestinal symptoms. Critical actions involve airway control, seizure control, and adequate fluid hydration. The toxidrome of this ingestion is typically transient and management is primarily supportive.

## Introduction and background

Composition C4 is a plastic explosive used in military operations. C4 was used during the Vietnam War as part of demolition blocks; today, it is commonly used in both military and civilian settings for demolition and flares. C4 is composed of RDX (91%), dioctyl sebacate (5.3%), polyisobutylene (2.1%), and mineral/motor oil (1.6%) [1]. RDX (Cyclonite) is the primary compound responsible for the explosive nature of C4 and has been utilized due to its malleable nature, which allows it to be molded into any desired shape and redirect the direction of the resulting explosion [2]. In the U.S. military, a wide variety of soldiers are exposed to this compound on the spectrum of basic training to combat deployments. Today, it is often used as an explosive for demolition and metal cutting, in underwater operations, and is found in claymore mines [3-4].

In the military, it became common knowledge that the ingestion of a small amount of C4 produces a similar “high” as that of ethanol, often leading to its consumption by soldiers [4]. The toxic effects of C4 were first described in workers who were exposed to chemical dust when handling and packing the material [4]. Several case reports have identified soldiers and military personnel who ingested C4 with multiple medical complications [5-8]. In each of these reports, patients presented with generalized seizures, hyperreflexia, nausea, and vomiting [5-8]. Multiple routes have been noted, including inhalation, skin contact, and ingestion; yet, ingestion seems to have the most severe consequences [4]. After ingestion, RDX is rapidly absorbed, reaches a peak concentration in four hours, and is eliminated in 48 hours [9]. It can cause central nervous system (CNS), renal, and gastrointestinal (GI) toxicity. In addition, some research has shown that RDX’s mechanism of action may be involved with the central nervous system [10-11].

Familiarity with and recognition of C4’s effects is important for the emergency medicine provider. This literature review addresses how to recognize the overall presentation of C4 intoxication and discuss the management of these patients in the hospital.

Objective

This literature review is intended to provide a broad overview of cyclonite/RDX exposure in humans to provide the physician with a general understanding of the clinical effects of exposure and their management considerations.

## Review

Methods

The authors searched MEDLINE and Google Scholar for articles using the keywords “RDX,” “hexahydro-1,3,5-trinitro-1,3,5-triazine,” “CAS number 121-82-4,” “cyclonite,” “C4,” “composition 4,” “ingestion,” “explosive,” and “toxicity” for this literature review. Case reports, case series, animal studies, clinical guidelines, and reviews were included. The literature search was limited to articles published in English with a focus on emergency medicine and clinical presentation with no geographical limitations. Emergency physicians with experience in the critical appraisal of the literature decided which studies to include for the review by consensus.

Results

A total of 162 articles were found on the initial search after the removal of duplicate articles. 140 articles were excluded based on exclusion criteria, including if studies were not full text, with only the abstract present, if animal studies did not propose mechanisms of action, if there was no clinical relevance, or if there were studies that only described the general toxidrome. Three full-text articles were not included due to not being in English. A total of 19 articles were selected for inclusion. Of these 19 articles, 10 were case reports/series, five were animal studies describing the mechanism of toxicity, three described management, and one was an army manual on explosives.

Animal studies/Pathophysiology

Several animal studies provide insight into the pathophysiology behind the adverse side effects of RDX. One animal study using rats utilized microRNA and mRNA profiles to help quantify biomarkers and propose mechanisms for RDX toxicity. This study described RDX’s role in the modification of immune and inflammatory response microRNA and genes and how these modifications potentially explain the neurotoxicity associated with RDX exposures [12]. Bannon et al. also utilized rats to show that RDX is quickly absorbed into brain tissue, with a peak effect within 3.5 hours with changes in gene expression and a correlating unregulated metabolism in the liver that showed below- detection limit levels of RDX at 48 hours [9]. Quinn et al. reported that increasing doses of RDX were associated with higher mortality rates in Northern Bobwhites [13]. In this study, the lowest-observed-adverse-effect level was determined to be 8/mg/kg/d. In another study with Crouse et al., a similar correlation was determined with Fischer rats that showed a no-observed-adverse-effect level of 4 mg/kg/d [14]. However, the side effects preceding death included tonic-clonic convulsions and weight loss secondary to GI toxicity [13]. A follow-up neurotoxicogenomic study executed with a cDNA microarray identified that Northern Bobwhites that exhibited RDX-induced seizures had 20 times more RDX in the brain tissue as compared to those of the non-seizing birds with a non-lethal dose. It was hypothesized that RDX elicited seizures by the inhibition of neuronal cell repolarization with regards to the post-action potential, which involved transcripts that encode Na+-K+ ATPase and calmodulin, leading to heightened neuronal excitability and hence seizures [15].

Further research has shown that RDX has direct effects on the CNS. Burdette et al. found that RDX affected the amygdala in rats, resulting in a seizure [16]. The amygdalas of these rats showed accelerated kindling or increased electrical stimulation, suggesting they may participate in RDX-induced seizure susceptibility [16]. In another study with rats, Williams et al. reported that RDX directly binds directly to γ-Aminobutyric acid type A (GABAA) receptors, causing a reduction in GABAergic inhibitory transmission, which results in seizures [11]. In essence, the toxic effects seen from RDX exposures may be secondary to various mechanisms, including gene modification and GABA receptor inhibition.

Clinical presentation

The presenting signs and symptoms of C4 toxicity range from headache, fever, nausea, and vomiting to lethargy and generalized seizures [8,17-19]. GI symptoms can present several hours after ingestion and usually occur earlier than CNS symptoms [8,16]. In order of presentation, CNS symptoms usually begin with confusion, hyperirritability, myoclonic seizures, and generalized tonic-clonic seizures [4,7,19]. Other findings that have been observed include tachycardia, hematuria, proteinuria, and oliguria [9,17-18].

RDX ingestion has been associated with several laboratory abnormalities, including leukocytosis, transaminitis, metabolic acidosis, elevated blood urea nitrogen (BUN) and creatinine, increased creatine kinase (CK), and myoglobinuria [6-7,17-18]. KüçükardalI et al. described three patients with severe metabolic acidosis not responsive to therapy that required hemodialysis; however, this was only seen in some case presentations and not in all patients [16]. A chest X-ray may reveal signs of pneumonitis secondary to gastric aspiration due to the effects of polyisobutylene, another component of C4 that should be managed appropriately [8,17-19].

The initial assessment of all these patients includes a focused history and physical examination. Co-ingestions should be considered, as several reports describe military personnel who have combined C4 with ethanol when presenting with C4 toxicity [17-18]. Hyperreflexia in the setting of acute RDX ingestion has been reported, but overall examination findings are nonspecific [4,17-19]. Providers must consider C4 in soldiers who are training with exposure and/or access to C4 and present with sudden onset epileptiform activity.

Management considerations and recommendations

The management of patients who present with C4 ingestion focuses on symptomatic and supportive treatment. It is reasonable to manage them in an algorithmic fashion, beginning with airway maintenance and the prevention of gastric aspiration. The electrocardiogram (ECG) may show potential arrhythmias from polyisobutylene; however, no arrhythmia specific to RDX toxicity has been identified [8]. In addition, several studies demonstrate that RDX is non-dialyzable and has a slow absorption rate [8-9,17-18]. With this consideration, decontamination such as adsorption with activated charcoal is reasonable [8].

Seizures have been consistently reported with RDX ingestions. Standard doses of lorazepam and diazepam have been effective in terminating initial grand-mal seizures [8,18-19]. Given RDX’s proposed effects on GABA receptors, benzodiazepines should be the first-line treatment to abort seizures associated with this ingestion. One report identified the use of phenytoin after the initial seizure presentation that was used throughout the patient’s hospital admission [7]. The efficacy and necessity of continued antiepileptic medication after the initial RDX-induced seizure is uncertain. If available, a serum RDX level would be helpful to correlate clearance and overall recovery.

After the initial management of hemodynamic abnormalities and seizure control, patients require judicious fluid support and the monitoring of urine output given the potential renal complications of hematuria, proteinuria, and oliguria that have been reported after ingestion [8,17-18]. Admission to the medical intensive care unit (ICU) with telemetry and monitoring (ECG, noninvasive blood pressure, pulse oximeter) has been reported [8,17-19]. Reports suggest that patients do not develop subsequent seizures after initial presentation thus EEG monitoring is unlikely to change management [7,17-18].

## Conclusions

C-4 is a plastic explosive used in military units and in select civilian settings. There are few reports or studies that identify the toxicities of C4. When ingested, C4 results in serious CNS effects such as generalized seizures in addition to renal and GI symptoms. Based on previous reports, the toxic presentation is transient and treatment is supportive. Critical actions include airway maintenance, seizure control, and fluid resuscitation. The use of RDX plasma concentrations can confirm ingestion but is unlikely to change the patient’s management plan. Although a rare occurrence, C4 toxicity should be considered in the workup of an undifferentiated seizure in a healthy patient, especially in populations that use or have access to explosives.
